# Exercise Thermal Sensation: Physiological Response to Dynamic–Static Steps at Moderate Exercise

**DOI:** 10.3390/ijerph18084239

**Published:** 2021-04-16

**Authors:** Qinghao Xu, Lin Chen, Hao Chen, Bart Julien Dewancker

**Affiliations:** 1 School of Electric Power, Civil Engineering and Architecture, University of Shanxi, Taiyuan 030000, China; chen31415926353@163.com; 2 Graduate School of Environmental Engineering, The University of Kitakyushu, Kitakyushu 8080135, Japan; bart@kitakyu-u.ac.jp; 3 School of Mathematical Sciences, University of Shanxi, Taiyuan 030000, China

**Keywords:** dynamic–static steps, exercise thermal sensation, physiological parameter, regression model, thermal alliesthesia

## Abstract

The study of exercise thermal sensation is more difficult than that of static thermal sensation in the human body. This work’s main purpose was to examine specific changes in human physiological parameters and subjective perceptions during the exercise process, especially around dynamic–static steps, and to assess exercise thermal sensation. Experiments were conducted in a climate chamber. A total of 16 subjects participated in two activities of different intensities on a treadmill, namely at 4.5 km/h and 6 km/h. The experimental procedure was set to static–dynamic–static. Skin temperature (T_sk_), oral temperature (T_or_), heart rate (HR), heart rate variability (HRV) parameters, and electrodermal activity (EDA) were measured at fixed time points, and thermal sensation values, thermal comfort values, and sweat feeling index were collected. The results showed complex changes in physiological indicators around the dynamic–static steps. Some important physio-logical indicators can be used as valid parameters for exercise thermal sensation models, such as T_sk_, T_or_, and EDA. This study highlighted that prediction models using average change and rate of change of measurements were better than using the original measurements. Our findings suggest that the exercise thermal sensation prediction models should be constructed according to the dynamic–static state and that psychological factors cannot be ignored.

## 1. Introduction

With the development of technology, indoor temperature regulators such as air conditioners and electric heaters have become essential appliances in homes and provide year-round temperature adjustments within a comfortable range [1,2,3]. However, the thermal sensation and the comfort of the human body often change significantly because of changes in environmental conditions or as a result of different activities in the building. For example, in a fitness center, if the indoor thermal environment is set to a comfortable or slightly warmer level when people are in a quiet state, the thermal sensation caused by exercise is intensified, occupants will sweat sooner and fatigue earlier, and their comfort will be reduced. This unsuitable environment may cause extra bodily energy expenditure when performing high-intensity exercise. Therefore, it is important to study the exercise thermal sensation in depth to control the thermal environment effectively.

From the viewpoint of heat transfer, the various factors that affect human thermal comfort and thermal sensation are influenced by the heat transfer methods such as evaporation, convection, and radiation between the body and the surrounding thermal environment, which affects human thermal comfort and thermal sensation. There are two common directions in which human thermal sensation and thermal comfort are often assessed and discussed. One is to study thermal sensation by exposing the body to specific thermal environments (e.g., different temperatures, humidity, thermal radiation, and air velocities). The other is to establish an intrinsic relationship with thermal sensation from the viewpoint of human physiological parameters. This work falls into the second category. Choi et al. [4] investigated the possibility of using human skin temperature to assess thermal sensation. Xiong et al. [5] studied the physiological and biochemical responses of humans to temperature steps and showed that oral temperature, skin temperature, heart rate, and heart rate variability are sensitive to temperature step changes. Luo et al. [6] described the modified equation of metabolic rate change, which can provide a useful reference for thermal comfort research. These studies above were conducted based on individuals with a low activity level (sitting). However, people are not always static indoors, and different levels of activity affect the human thermal sensation and thermal comfort.

Li et al. [7] considered that the temperature of the skin on the wrist and its time lag and heart rate could be used to estimate the human thermal sensation with high accuracy for different activities. The human body typically takes 3–5 min to reach a new metabolic level after walking and 4–5 min to return to a normal sedentary state after exercise. Choi et al. [8] posit that the heart rate is significantly related to the human metabolic rate and may be a potential parameter that directly (or indirectly) affects the cause of thermal comfort. The purpose of the research by Wang et al. [9] was to understand better the real thermal sensation of individuals performing moderate activities. They found that the thermal sensation of the subjects was related to sweat activity. As the activity intensified, the subjects expected higher air velocity. Fanger noted that skin temperatures and sweat rates for people in thermal comfort depend upon activity level, as assumed by the famous PMV equations [10]. These studies all showed that physiological parameters are closely related to exercise thermal sensation.

Diyi Tan et al. [11] studied the thermal sensation of people who alternated between sedentary exercises and walking on a treadmill at 20 and 25 °C. The experimental results showed that when the thermal sensation failed to reach a steady-state, PMV was not suitable for predicting the thermal sensation of active individuals. Gagge et al. [12] studied the relationship between activity levels and human thermal sensation and thermal comfort under different working conditions. They found that thermal discomfort was principally related to skin sweating and conductance and was affected either by air temperature and metabolism or by both skin and rectal temperature. Gagge conducted a study on four male subjects, and kept them under different temperature conditions, gradually changing from resting to different activity levels. Studies have shown that subjects reached new and stable levels of thermal sensation and thermal comfort 20–30 min after a change in activity status. Many of the above studies involved changes in exercise state. However, it should also be noted that thermal sensation adjusts and changes accordingly after a change in exercise status. However, over a relatively short period of time after a change in exercise status, it is unclear how detailed changes in physiological indicators are related to changes in thermal sensation, and whether these detailed changes affect the prediction model.

This work attempted to investigate the relationship between physiological parameters and the exercise thermal sensation. These parameters include skin temperature, oral temperature, heart rate, heart rate variability, and electrodermal activity. The aim was to use physiological indicators to assess exercise thermal sensation more accurately. The specific objectives were to first investigate physiological parameter changes throughout the exercise (static–dynamic–static) by experiment, especially around dynamic–static steps. Afterward, to explore and constructed mathematical relationships between these physiological indicators and thermal sensation (TSV). Finally, the advantages and disadvantages of different assessment models are compared.

## 2. Methods

### 2.1. Subjects

Experiments were conducted with 16 subjects recruited from cities in northern China using physiological measuring instruments and subjective questionnaires.

Table 1 shows basic information, including age, gender, body mass index (BMI) [13], and body surface area (As) [14]. All subjects wore regular clothes (underclothes, long-sleeved T-shirts, thin trousers, socks, and sneakers) with clothing insulation of 0.8 clo. Clothing insulation values were calculated using the ASHRAE standard 55 [15].

To avoid the influence of age and social background on the results, the subjects were all college students from the same province. Subjects were required to be healthy and free of underlying diseases and were not allowed to take drugs before or during the experiment. Vigorous exercise and overeating were not allowed within 24 h of the test. Subjects were not allowed to eat or drink for 2 h before the test. All protocols were approved by the University Ethics Committee (approval number SXULL2019069). Before participating in the experiment, verbal and written informed consent was obtained from each subject.

### 2.2. Experimental Environment

**Figure 1:**
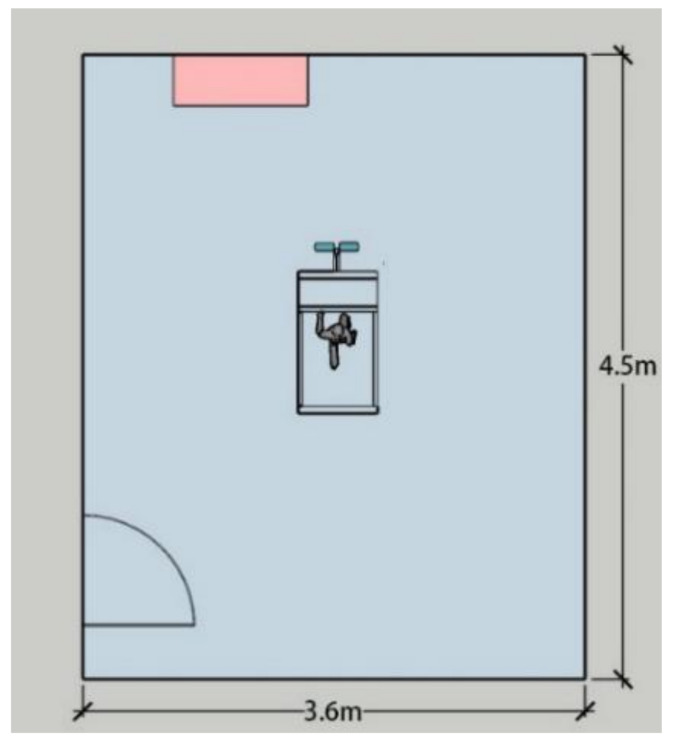
Climatic chamber diagram.

The experiment was conducted in Taiyuan—a typical cold region in northern China—in October (a comfortable season). The test was performed in a climate chamber (Figure 1). The indoor environment had a temperature of 26 °C, a relative humidity of 60%, an average radiation temperature equal to the indoor temperature, and air velocity *v*_a_ < 0.1 m/s.

### 2.3. Experimental Measurements

#### 2.3.1. Measurement of Physiological Parameters

Physiological parameters were monitored throughout the experiment. Special attention was given to changes in the exercise state changes (dynamic–static steps). The physiological parameter measuring instrument was portable, and the indicators of the device are shown in Table 2. The arithmetic mean of all data collected within 5 s before and after the measurement of each physiological parameter was calculated to improve the validity of the data.

#### Skin and Oral Temperatures

Skin temperature is closely related to the human thermal sensation and can be used as an essential indicator in human activity experiments. When performing skin tempera-ture measurements, the skin of the measurement area was cleaned or wiped with alcohol, and the sensing probe was attached to the surface with vapor-permeable surgical tape. The sensor probe was firmly attached to the skin. The skin temperature was calculated by taking the data from the seven body parts automatically recorded by the temperature sensor and inserting the values into Equation (1) [16]. The oral temperature was used as the core temperature in this experiment. Xiong et al. [17] used oral temperature and skin temperature as markers of the thermal metabolic system, and oral temperature was used as an indicator of core temperature. Zhang et al. [18] used auditory canal temperature as the core temperature in the dynamic test. Zhai et al. [19] and Gao et al. [20] measured core temperature using telemetry pills. Chappuis et al. [21] found that sublingual temperature (T_or_) was somewhat higher than the tympanic membrane temperature (T_ty_) at 25 and 30 °C at rest. During exercise, the difference between T_or_ and T_ty_ became smaller. There was a good correlation between these two measurements during the 90 W exercise. Furthermore, since it was challenging to achieve measurement of rectal temperature, tympanic temperature, etc., in dynamic testing, and considering future practicality, we finally chose to measure oral temperature, which was the most convenient and feasible method to measure body temperature in this experiment. When measuring the oral temperature, the sensor probe was sterilized and placed under the tongue. The subject was required to breathe through the nose during the whole process to avoid errors caused by mouth breathing during exercise. Breathing through the nose satisfies the oxygen demand of the body when the exercise intensity is moderate, which was the case in this experiment. Ear canal or anal temperatures are recommended for high-intensity exercise testing.

(1) $$T_{\mathrm{sk}}=0.07T_{\mathrm{forehead}}+0.35T_{\mathrm{chest}}+0.14T_{\mathrm{lowerarm}}+0.05T_{\mathrm{handback}}+0.19T_{\mathrm{thigh}}+0.13T_{\mathrm{lowerleg}}+0.07T_{\mathrm{instep}}$$

#### HR and HRV

The heart rate (HR) reflects the activity of the heart. Heart rate variability (HRV) is a non-invasive method for assessing the effects of the autonomic nervous system [22]. HRV consists of time and frequency domain metrics. The frequency domain is a method of separating the waveforms in the heart rate graph according to different frequencies and analyzing and researching in a specific frequency range (this experiment used LF/HF as the primary indicator). The time domain method analyzes the time domain heart rate and heartbeat interval within the test and studies its change over time [23]. The heart rate variability index in time domain analysis primarily reflects the function and state of the parasympathetic nerve. This experiment used standard deviation of the NN (R–R) intervals (SDNN) and root mean square of the successive differences (RMSSD) as the primary indicator. SDNN reflected the total change of heart rate variability and the total activity of the sympathetic and vagus nerves. RMSSD was primarily used to evaluate the cardiac vagus nerve regulatory function in the high-frequency field and is commonly employed as a parameter for assessing parasympathetic nervous system activity [22,24]. According to HRV analysis theory, LF is related to sympathetic and parasympathetic nerve activity, and HF indicates vagus nerve activity. Therefore, LF/HF indicates the dominant activity and potentially affects thermal comfort by acting on the vagus and sympathetic nerves of the human body [16]. This experiment used the photo plethysmography (PPG) method of measurement, which uses photoelectric technology to detect blood volume changes in human tissue to calculate pulse waves. During the experiment, the subject clipped the sensor’s black ear clip to the ear-lobe, and the strap is attached to the arm or wrist. The earlobe needs to be gently massaged before the experiment to promote blood flow in order to prevent any noise or interference with the acquisition signal. The connection cable is attached to the garment’s collar to avoid shaking the instrument due to movement.

#### Electrodermal Activity (EDA)

EDA is the physiological response of an individual related to the sympathetic nervous system and may be a useful indicator of the sympathetic branch activity of the autonomic nervous system, because sweat glands are innervated by sympathetic nerve activity [25,26]. EDA is an autonomous change in the electrical characteristics of the skin caused by sweat secretion [27]. EDA can also be used as an indicator of sweat secretion. The skin area to which the sensor electrodes need to be connected is wiped or cleaned before performing EDA measurements. Conductive paste is then applied to the skin area, and the two sensor electrodes are attached separately to the skin area where the conductive paste was applied.

#### 2.3.2. Subjective Selection

**Figure 2:**
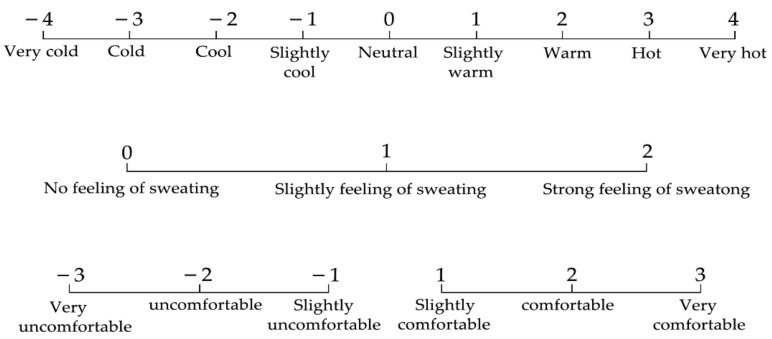
Selection scale for thermal feeling, thermal comfort, and sweat feeling indices.

These indices include thermal sensation (TSV), thermal comfort (TC), and the sweat feeling index (SFI) (Figure 2). The thermal sensation was investigated according to the ASHRAE Thermal Sensitivity Scale [28], and subjects answered questions regarding thermal sensation on a 9-point scale (ASHRAE 7-pt scale with very hot and very cold added as endpoints), during the experiments. Thermal comfort rating scores range from −3 to +3. The levels of SFI are 0, 1, and 2 [9]. Fatigue was evaluated using a Japanese subjective fatigue checklist, which consisted of 25 items and was divided into five subtypes [29]. Participants answered each item using a 5-point discrete scale from +1 (none) to +5 (extremely severe).

### 2.4. Experimental Procedure

**Figure 3:**
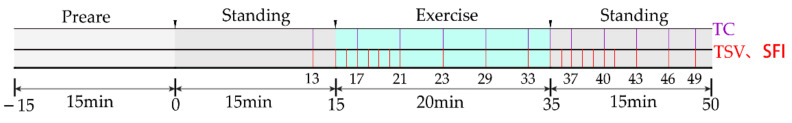
Experimental procedure.

The experiment was conducted in October 2018 and was divided into two sets (V_I_ and V_2_). Each subject was required to participate in both sets of experiments, and the procedure was identical in both sets, differing only in treadmill speed. Each subject participated in the tests at intervals of more than 72 h to ensure adequate acclimatization to the experimental conditions, to prevent biases in the results owing to any one set of the experiment. Before and after the experiments, we ensured that all measurement equipment was carefully calibrated. We assured that the following preparations were performed before the test:

In the preparing phase, subjects had 15 min to change clothes, learn how to make a selection, wear physiological sensors, and avoid entering the chamber with an elevated metabolic rate. Each set of experiments lasted 50 min and was divided into three phases (Figure 3). In the first phase (0–15 min), the subjects stood still in the climatic chamber. In the second phase (15–35 min), the subjects walked for 20 min on a treadmill at a certain speed (speeds in the two sets of experiments were v_1_ = 4.5 km/h and v_2_ = 6 km/h). In the third phase (35–50 min), the subjects stopped exercising and continued to stand for 15 min. A sitting position may wrinkle or fold clothing and yield different clo values. There-fore, the standing posture was maintained to avoid the formation of an air layer between the clothing layers. The ASHRAE Standard 55-2017 table [30] and the study by Zhai et al. [31] were used to estimate the metabolic rate value. The activity intensities of V_1_ and V_2_ were approximately 3.4 and 4.9 met, respectively, and were considered medium intensity exercise. Subjects’ physiological parameters and subjective perception questionnaires were completed at certain time points during the experiment.

As shown in Figure 3, thermal sensation and SFI selection were conducted during the experiment at the 13th, 15th, 16th, 17th, 18th, 19th, 20th, 21st, 25th, 29th, 33rd, 35th, 36th, 37th, 40th, 43rd, 46th, and 49th minutes (red 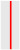 ). The 12 thermal comfort questionnaire times (violet 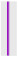 ) were at the 13th, 17th, 21st, 25th, 29th, 33rd, 35th, 37th, 40th, 43rd, 46th, and 49th minute. The exercise was stopped at the 35th minute. The questionnaire of thermal sensation and thermal comfort was commenced 10 s after the exercise to study the immediate subjective changes in the human body.

### 2.5. Data Processing

Raw data were input into Excel 2019 for organization and sorting. All data descrip-tion analysis was performed in Origin 2018, including data splitting and summarization. In this study, the data were described through line charts and histograms, and a boxplot was used to determine the outliers. The differences and correlations of the data were stud-ied with IBM SPSS Statistics 25. The Shapiro–Wilk method was used to conduct the nor-mality test; the sample T-test was performed on the test V_1_ and V_2_ data. The Pearson cor-relation coefficient was calculated, and the double-tail test was performed. The objective and subjective indexes were quantitatively analyzed by a stepwise regression algorithm; the model was established, and a significance test was carried out. The stepwise regres-sion method is a multiple linear regression analysis method. The stepwise regression al-gorithm was adopted to fit the regression model. The selection of independent variables was determined automatically by the stepwise algorithm. In each step, a variable was considered for addition to or subtraction from the set of explanatory variables based on some prespecified criterion. Statistical analyses were performed at the 95% significance level.

## 3. Experiment Results

### 3.1. The Impact of Dynamic–Static Step Changes on Physiological Parameters in Exercise

#### 3.1.1. Skin and Oral Temperature

**Figure 4:**
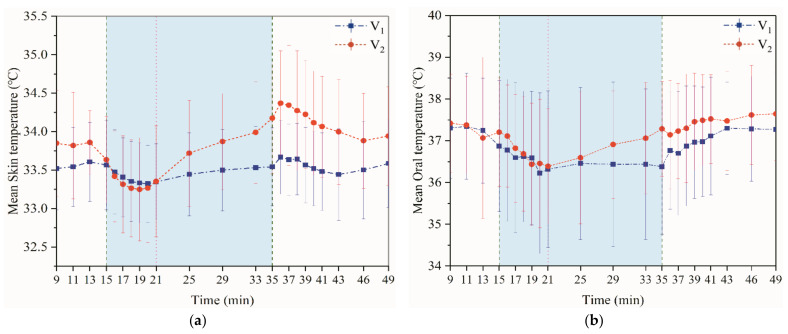
Multiple lines of mean skin and oral temperature (95% CI) by time and phase. (**a**) Mean skin temperature; (**b**) mean oral temperature. (V_1_: 4.5 km/h; V_2_: 6 km/h).

Figure 4a shows skin temperature over time. In the early stages of exercise, the skin temperature decreased slightly. It was important to note that the skin temperature overshoots at 36 min. The human body indicators in exercise fluctuate widely when compared to the static state.

Both oral and skin temperatures reflect the function of the thermal metabolic system of the body. Figure 4b shows that the oral temperature was maintained at approximately 37.2–37.4 °C before exercise. At the beginning of the exercise, the oral (core) and skin temperatures showed a downward trend. After the end of the exercise, the core temperature continued rising and was practically the same as the initial temperature. At the end of the exercise, overshoot was also observed. The oral temperature continued to rise until near the initial temperature.

#### 3.1.2. HR and HRV

**Figure 5:**
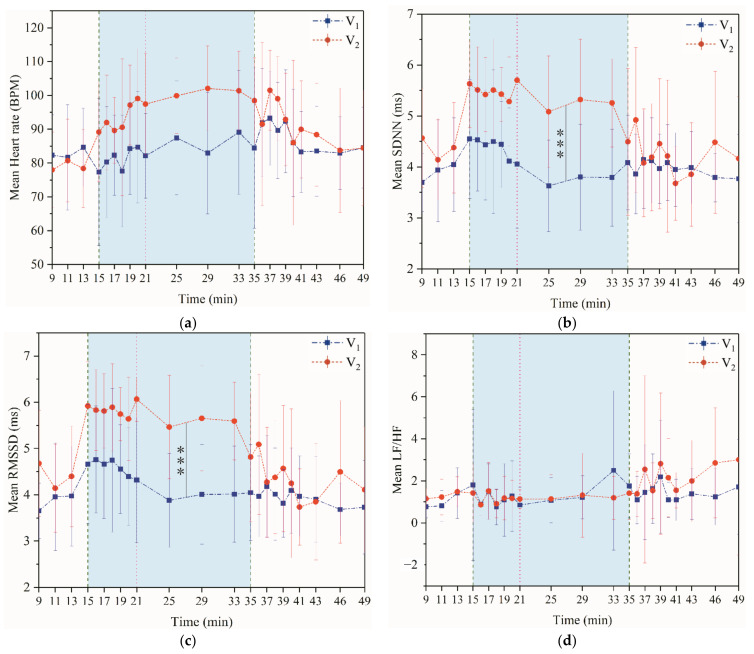
Multiple lines of mean HR and HRV (95% CI) by time and phase. (**a**) Mean heart rate (**b**) Mean SDNN (**c**) Mean RMSSD (**d**) Mean LF/HF.

Figure 5a shows that the heart rate before exercise is practically maintained at 75–80 BMP. The heart rate began to fluctuate at the start of the exercise and was higher in the V_2_ stage than in V_1_. The upward rush of the heart rate within 2 min after the exercise is a phenomenon that becomes more obvious as the exercise intensity increases. This trend was also found in the LF/HF index changes.

Figure 5b–d shows the changes over time for each indicator in the HRV. The change patterns of the time domain indicators were similar. At the beginning of the exercise, the time-domain index increased rapidly. During the exercise, standard deviation of the NN (R-R) intervals (SDNN) and root mean square of the successive differences (RMSSD) trended downward and recovered to the pre-exercise levels with a more significant drop after the exercise. It shows that the parasympathetic tone gradually decreases, indicating that the comfort level progressively worsens. The values of SDNN and RMSSD in the V_2_ experiment during exercise were significantly higher than V_1_ (*p* < 0.001, it was marked with “*” in Figure 5b,c. It showed that the human body was more uncomfortable in the V_2_ experiment. A study by Spring et al. [32] showed that the subjects experienced a decrease in RMSSD and HF during exercise compared to in a resting state and that the sympathetic-vagal nerve was imbalanced. Maraes et al. [33] found that RMSSD and HF decreased after walking, while LF increased. The increase in LF/HF and the decrease in RMSSD after exercise in this test were consistent with the above study. There was no significant change before and during exercise in the frequency-domain indicator LF/HF, indicating that the active degree of difference between the sympathetic and parasympathetic nerves was the same; thus balancing the entire autonomous audit system remains unchanged. However, the data visualization revealed that the index has some relationship with thermal sensation and thermal comfort.

#### 3.1.3. Electrodermal Activity

**Figure 6:**
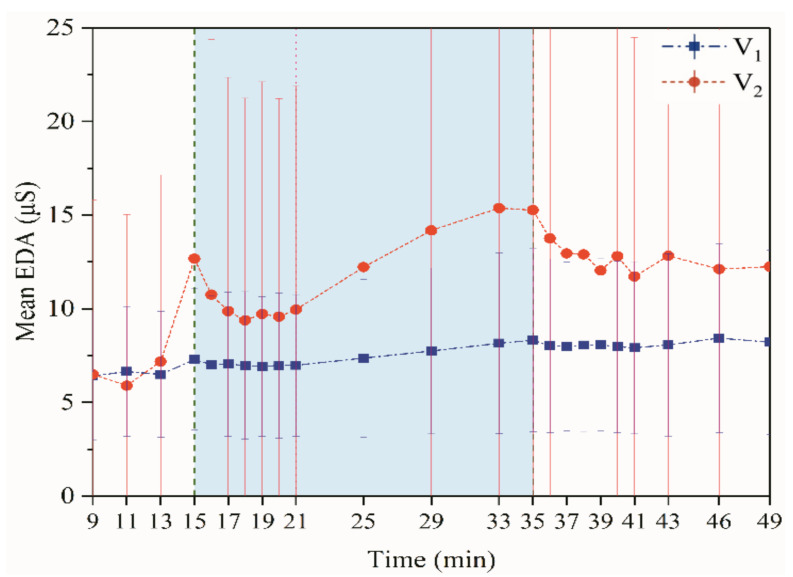
Multiple lines of mean electrodermal activity (EDA) (95% CI) by time and phase.

The EDA value suddenly changed at the moment when the exercise started (Figure 6). This phenomenon is more evident in the V_2_ experiment and is a physiological stress response because of the sudden changes in exercise status. The value was very sensitive because the EDA sensor probe is fixed to the tip of the index finger, the part of the human that first sweats after being stimulated by external stimuli or sudden changes in mood. Higher EDA can reflect the stimulation of sympathetic nerves by external stressors, which was the result of feedback from the autonomic nervous system [34]. This mutation result is consistent with the time-domain indicators (SDNN and RMSSD) in the HRV. After the stress level was lowered, the EDA index decreased and returned to normal levels. However, the EDA index of the V_2_ experiment was generally higher than V_1_, indicating that the amount of sweat in the V_2_ test is higher than in V_1_. By the last stage of the exercise, the EDA index gradually increased with the increase in sweating and reached a peak at the end of the exercise. The sympathetic nerve was more active at this time, and the discomfort is stronger. The EDA index decreased after exercise in the V_2_ experiment but did not return to pre-exercise levels. Because this experiment was performed in a steady-state environment, the subjects exercised in situ and dressed in long-sleeved clothing and trousers to allow sweat to evaporate slowly. The EDA indicator takes longer to return to its initial value before the exercise.

### 3.2. The Impact of Dynamic–Static Step Changes on Subjective Sensation in Exercise

#### 3.2.1. Thermal Sensation, Thermal Comfort and SFI

**Figure 7:**
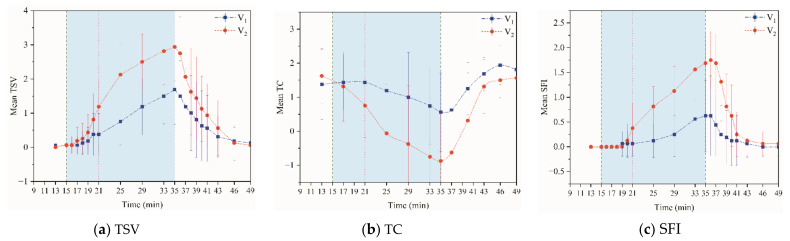
Multiple lines of mean TSV, TC and SFI by time and phase.

Figure 7a shows the change in the mean human thermal sensory vote (MTSV) throughout the experiment. Overall, V_2_ had a higher thermal sensation than V_1_. Over time, the metabolic rate of the body gradually increased, and the body heat load gradually increased, which made the thermal sensation increase progressively. TSV, TC, and SFI were asked about 10 s after the exercise stopped (35th min). The thermal sensation did not weaken immediately after the exercise stopped but instead reached its peak. Hence, at the moment when the continuous exercise was completed, the thermal sensation overshot. This phenomenon became more evident with the increased intensity of the activity.

Figure 7b shows the change in thermal comfort. Increased duration or intensity of the exercise caused thermal comfort to decrease gradually. The comfort vote was conducted 10 s after the exercise ended, at which time thermal comfort reached its lowest level. The thermal comfort at the end of the V_1_ experiment was higher than the beginning of the test, likely because of the thermal alliesthesia factor [35]. Based on daily experience, the human body generally feels comfortable and relaxed after the exercise. At the end of the V_2_ experiment, although the thermal comfort was lower than the beginning of the experiment, the curve showed an upward trend. If the experiment time was increased, the same results as V_1_ will likely appear. Thus, with increased exercise intensity, the recovery of thermal comfort will be slower and eventually higher than before exercise, a reminder that studying the thermal comfort of human movement must consider the effects of the thermal sensation and pay attention to thermal delight.

The SFI was practically 0 at the beginning of the exercise (Figure 7c), and a weak sweaty feeling appeared approximately 3 min after the start of the exercise. Within a minute after the exercise stopped, the sweaty feeling did not stop and experienced an overshoot similar to the change in thermal sensation. However, it should be noted that the overshoot of SFI slightly lagged behind the overshoot of thermal sensation. Additionally, the subjective sensation lags more than that of the EDA index. The sweat feeling after exercise took shorter to subside than it took to grow.

#### 3.2.2. Fatigue Index

**Figure 8:**
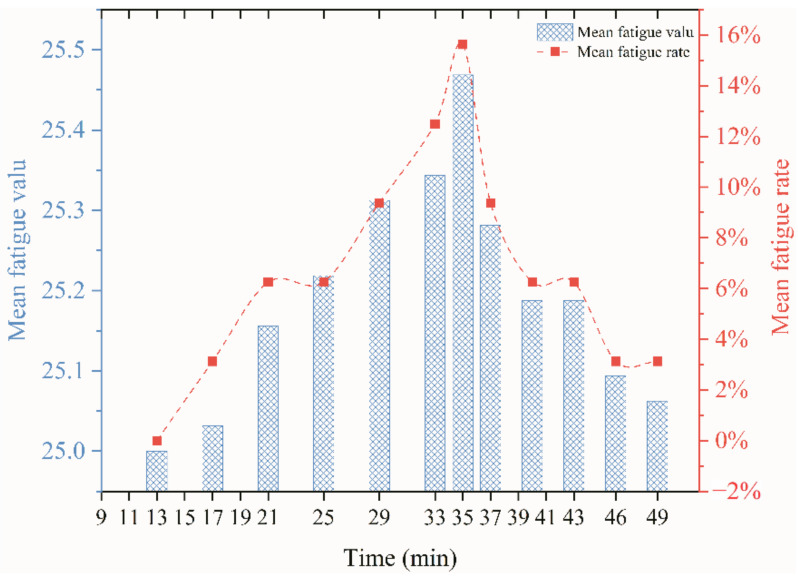
The biaxial plot of mean fatigue value and fatigue rate.

The left axis in Figure 8 represents the fatigue index, and the right axis represents the number of subjects who were fatigued as a percentage of the total. The highest fatigue value occurs after the exercise has stopped. The number of subjects suffering from fatigue at the same time was also the largest. Fatigue was relieved faster after exercise than it occurs during exercise. The number of subjects whose fatigue disappeared was higher than the number of subjects whose fatigue increased.

## 4. The Mathematical Relationships between Physiological Indicators and Subjective Perceptions in Dynamic-Static Step Processes

This study used a stepwise regression algorithm to quantitatively analyze subjective and objective indicators, establish a model, and perform a significance test. This study established models for the two exercise intensity experiments. The regression model in this article did not include the evaluation of TSV and TC before exercise.

### 4.1. Stepwise Regression Analysis between Physiological Indicators and TSV

The relationship between TSV and various physiological indicators under different exercise intensities, including T_or_, T_sk_, RMSSD, SDNN, LF/HF, and EDA, was investigated. Table 3 shows the stepwise regression results of TSV for T_or_, T_sk_, RMSSD, SDNN, LF/HF, and EDA in the V_1_ and V_2_ experiments. In the V_1_ experiment, EDA, T_or_, and T_sk_ remained in the model after calculation, F = 47.88 (*p*-value < 0.001), indicating that the model was statistically significant. The *p*-values of significance tests corresponding to EDA, T_or_, and T_sk_ are <0.001, <0.001, and 0.026, which means that the regression relationship was significant. EDA entered the model first and produced an R_2_ of 38.1%, indicating that EDA has an explanatory power of 38.1% for the dependent variable. T_or_ and T_sk_ entered the model next, and R_2_ increased by 48.1% and 3.81%, respectively. Thus, the ability of the model to interpret TSV reached 90%, and the model can express 90% of the information volume of the V_1_ data set. RMSSD, SDNN, and LF/HF were excluded from the model variable set due to insufficient significance. In the V_2_ experiment, after automatic selection, EDA, T_or_, and T_sk_ increased R_2_ by 52.2%, 24.8%, and 15.5%, respectively. The interpretation ability of EDA and T_sk_ of the thermal sensation gradually strengthened as exercise intensity increased, while the interpretation ability of T_or_ weakened. Equations (2) and (3) are the results of two experimental stepwise regression algorithms. EDA and T_sk_ have a positive effect on TSV, and Tor has a negative effect on TSV. Skin and oral temperature are relevant physiological indicators of the body’s thermal metabolic system and reflect, to some extent, an individual’s metabolic rate. The results also show that the parameters related to metabolic rate were still indispensable in the evaluation of the exercise thermal sensation.

(2) $$V_{1}:\;\mathrm{TSV}\;=-15.284+0.846\mathrm{EDA}\;-1.299T_{\mathrm{or}}+1.711T_{\mathrm{sk}}$$

(3) $$V_{2}:\;\mathrm{TSV}\;=14.010+0.325\mathrm{EDA}\;-2.446T_{\mathrm{or}}+2.189T_{\mathrm{sk}}$$

**Figure 9:**
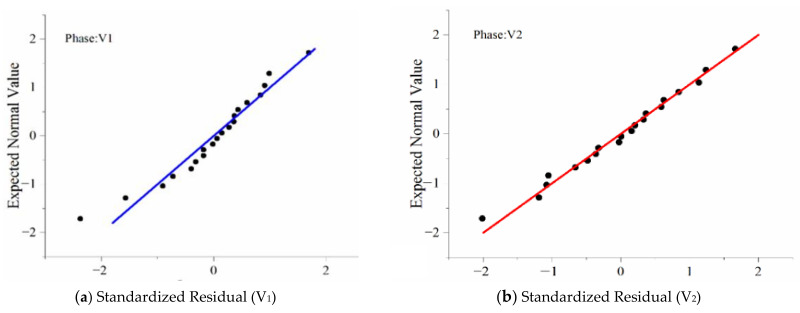

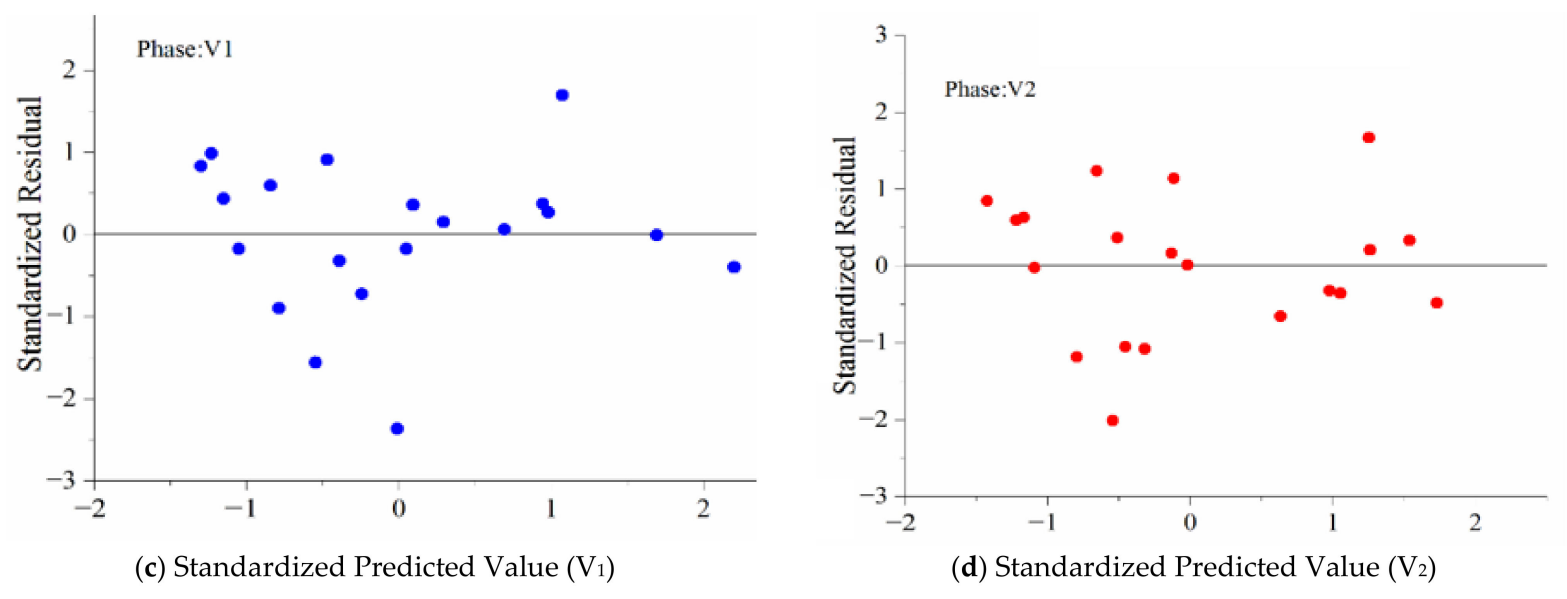
Normal Q-Q plot and scatterplot of the regression standardized residual of TSV.

Figure 9 shows that by analyzing the residual Q-Q diagram and scatter plots, the residual reveals the characteristics of “White Noise” and obeys the standard normal distribution.

### 4.2. Stepwise Regression Analysis between Physiological Indicators and TC

We further examined the relationship between TC and various physiological indicators, including T_or_, T_sk_, RMSSD, SDNN, LF/HF, and EDA. Table 4 shows the results of the stepwise regression. In the V_1_ experiment, T_or_ and T_sk_ remained in the model, and the model significance test *p*-value = 0.001, indicating that the model had statistical significance. The *p*-values for the significance test of the corresponding coefficients of T_or_ and T_sk_ were < 0.001 and 0.007, respectively, indicating that the regression coefficient was significant. T_or_ and T_sk_ increased the R_2_ of the model by 51.8% and 27.9%, respectively, increasing the model’s ability to interpret TSV to 79.7%. EDA, RMSSD, SDNN, and LF/HF were excluded from the model variable set due to insufficient significance. In the V_2_ experiment, EDA, T_or_, and T_sk_ remained in the model. Significance test *p*-values < 0.001 for EDA, T_or_, and T_sk_ produced coefficient significance test *p*-values of 0.044, <0.001, and 0.005, respectively, indicating that the model and coefficient were significant. The R_2_ corresponding to EDA, T_or_, and T_sk_ increased by 43.2%, 27.9%, and 18.8%, respectively. Equations (4) and (5) are the results of two experimental stepwise regression algorithms. T_or_ had a positive effect on TC, and T_sk_ and EDA had a negative effect on TC. With the increase of exercise intensity, EDA was still an essential parameter for predicting comfort.

(4) $$V_{1}:\;\mathrm{TC}\;=88.827+0.967T_{\mathrm{or}}-3.801T_{\mathrm{sk}}$$

(5) $$V_{2}:\;\mathrm{TC}\;=1.418-0.229\mathrm{EDA}\;+2.438T_{\mathrm{or}}-2.7T_{\mathrm{sk}}$$

**Figure 10:**
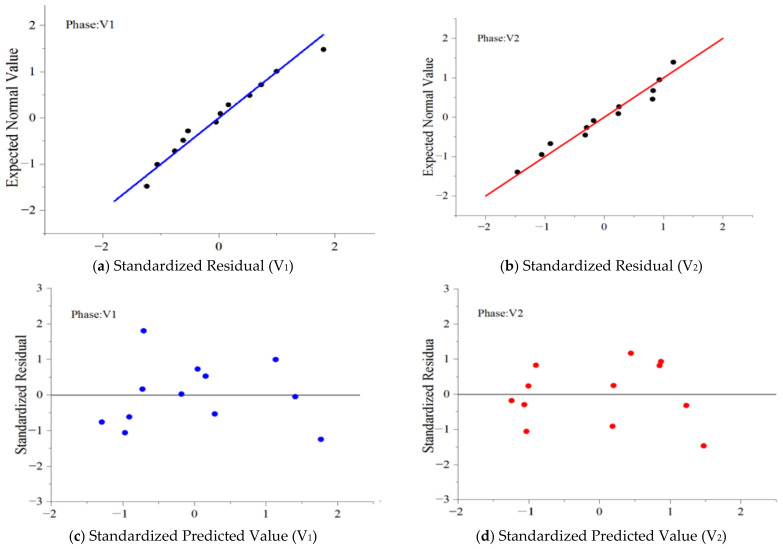
Normal Q-Q plot and scatterplot of regression standardized residual of TC.

Analyzing the residual Q-Q diagram and scatter plots revealed that the residuals had the characteristics of “white noise” and obeyed the standard normal distribution, further verifying the validity of the model establishment (Figure 10).

### 4.3. Average Change and Rate of Change of Physiological Parameters

**Figure 11:**
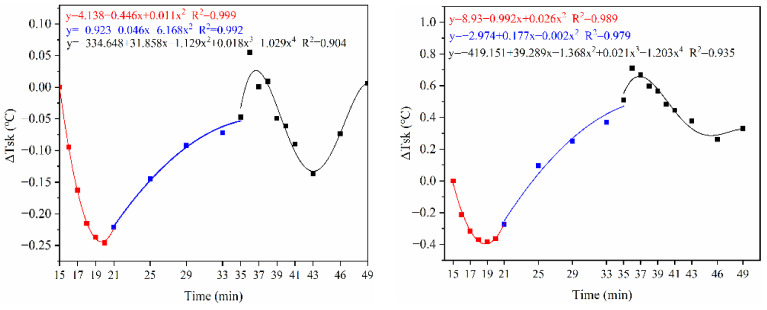

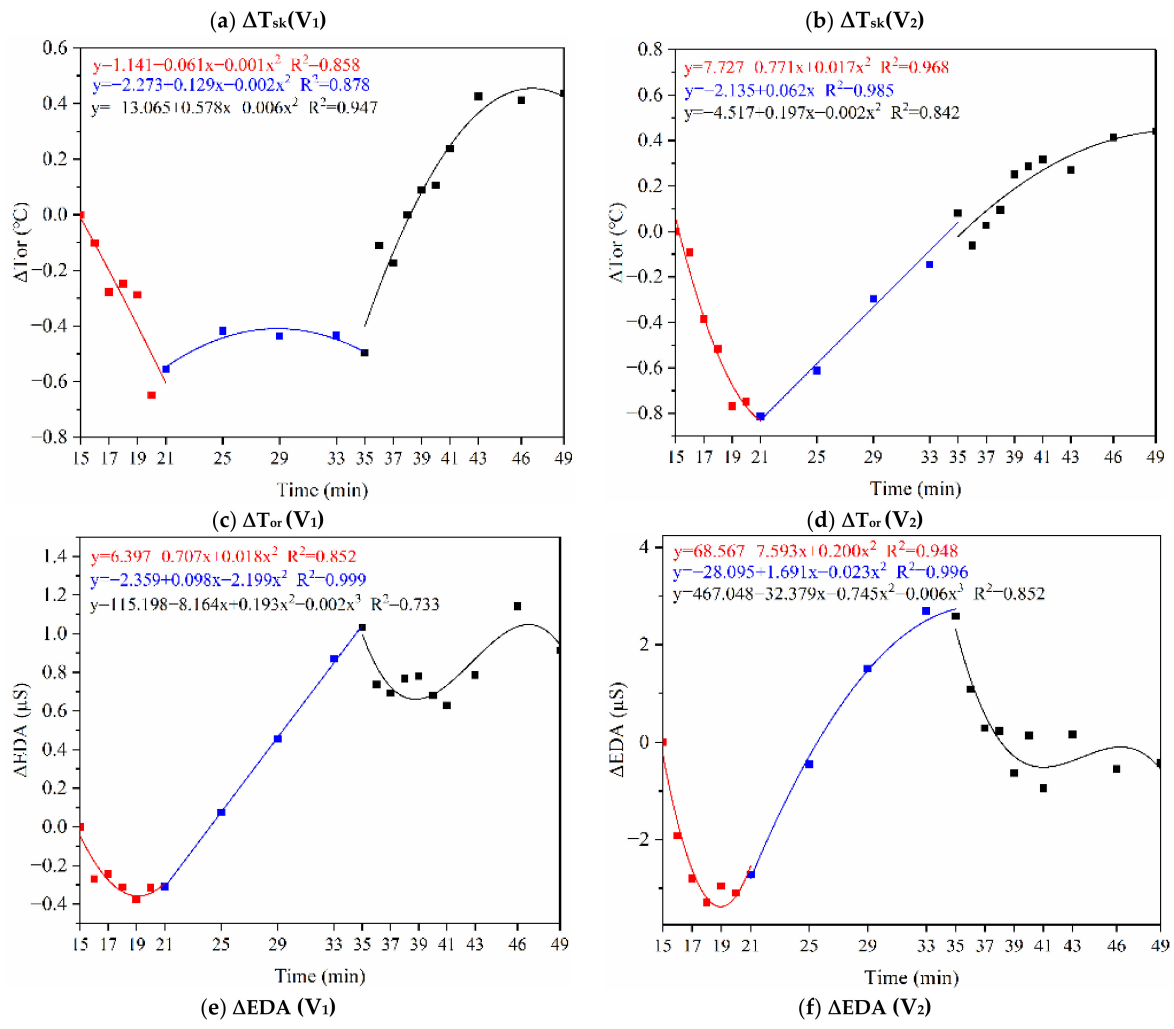
The average change of physiological parameters.

Figure 11 shows the average changes in skin temperature, oral temperature, and EDA. We compared the value of each testing moment with the initial value. That is, ∆T_sk_ = T(time(i)) − T (time (i = 15)), ∆T_or_ = T(time(i)) − T (time (I = 15)), ∆EDA = EDA (time(i)) − T (time (i = 15)). The average change of each physiological parameter in V_2_ was greater than in V_1_. i.e., skin temperature: V_1_ (−0.25 to 0.05), V_2_(−0.4~0.7); Oral temperature: V_1_(−0.65~0.45), V_2_(−0.8~ 0.47); EDA: V_1_(−0.4~1.2), V_2_(−3.4~2.9). When about 6 min since the exercise, the turning points of each parameter come to appear. After the end of the exercise, the function of change of each parameter over time was more complex.

**Figure 12:**
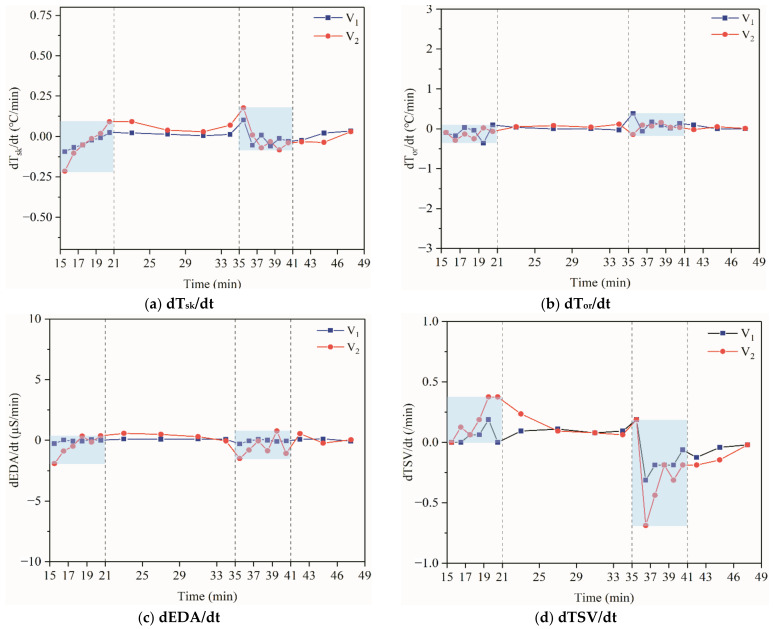
The rate of change of physiological parameters and TSV.

Figure 12 shows an example of the values corresponding to the rates of change for the three parameters and TSV. In general, the rate of change of each parameter fluctuates considerably during the first 6 min in the exercise and also in the early period of the post-exercise. As the exercise intensity increased, the rate of change was more significant. It was interesting to note that in 6 min after exercise, the rate of change becomes relatively stable (with a drift toward 0). Was the “symmetry” worthy of attention? That is, the timing of adjustment of physiological parameters and TSV were always similar after a mutation in metabolic levels(static-dynamic–static), which deserves further study. It means that after a sudden change in the activity state (dynamic–static steps), there was a dynamic process of adjustment of the physiological parameters. This adjustment process was more complex and lasts longer than the physiological adaptation caused only by changing the ambient conditions. Additionally, overshoot was observed for each rate of change. It was found that overshoot occurred in a short time after the exercise, while it was not noted at the beginning of the exercise.

**Figure 13:**
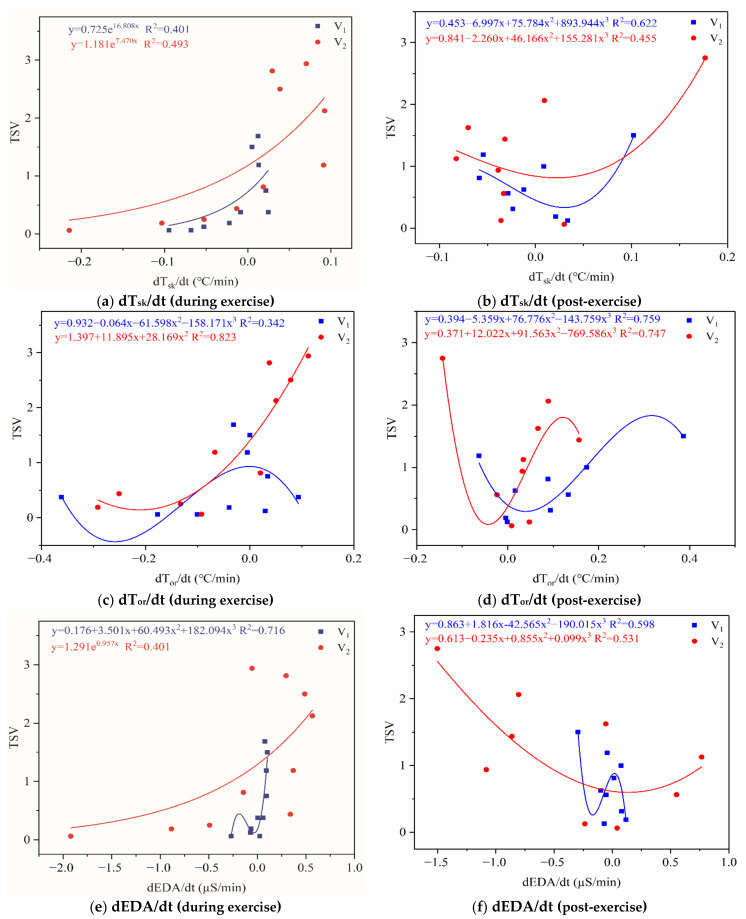
Thermal sensation as a function of the rate of change of physiological parameters.

Based on the above analysis, we selected several physiological parameters closely related to the exercise thermal sensation and examined the regression between the rate of change and thermal sensation. Figure 13 shows thermal sensation as a function of the rate of change of physiological parameters, separately for during and post-exercise. It shows that TSV was rapidly elevated after a positive rate of change in physiological parameters during exercise (i.e., a derivative greater than 0). That is, as the rate of change of physiological parameters accelerated during exercise, the TSV changed rapidly as well. It became more pronounced with increasing exercise intensity. From the regression equation, it can be seen that when the rate of change was equal to 0, TSV_2_ > TSV_1_. This meant that the higher the intensity of exercise during exercise, the greater the effect of the change rate of physiological parameters on TSV. When the rate of change of physiological parameters was 0 at post-exercise, the thermal sensation was all near the lowest point of the curve (0.3–0.8). In other words, thermal sensation returned to the comfort range when the physiological index was not changing over time. Besides, it can be observed that the curves appeared to be symmetrical during and post-exercise. That is, TSV was approximately similar to the changes in each physiological index in both phases, so it may be more reasonable to use the rate of change to predict the thermal sensation of exercise. It was then necessary to explore the use of the rate of change of physiological parameters to perform stepwise regression analysis of TSV.

### 4.4. Stepwise Regression Analysis of TSV Using Average Change and Rate of Change of Physiological Parameters

Stepwise regression continued to be used to examine the relationship between objective indicators and TSV, i.e., ∆T_or_, ∆T_sk_, ∆EDA, dT_sk_/dt, dT_or_/dt, and dEDA/dt. Table 5 and Table 6 show the results of the separate stepwise regressions during (DE) and post-exercise (PE) for the two sets of experiments. The Q-Q plots and scatters plots were carried out for these models. It was evident that the residuals showed “white noise” characteristics and obeyed the standard normal distribution, thus further verifying the validity of the model.

(6) $$V_{1}:\;\mathrm{DE}:\;\mathrm{TSV}\;=0.637+0.995∆\mathrm{EDA}\;+3.536{\mathrm{dT}}_{\mathrm{sk}}/\mathrm{dt}$$

(7) $$\mathrm{PE}:\;\mathrm{TSV}\;=0.883-1.748∆T_{\mathrm{or}}+1.025{\mathrm{dT}}_{\mathrm{or}}/\mathrm{dt}$$

(8) $$V_{2}:\;\mathrm{DE}:\;\mathrm{TSV}\;=0.896+4.225∆T_{\mathrm{sk}}-1.696∆T_{\mathrm{or}}$$

(9) $$\mathrm{PE}:\;\mathrm{TSV}\;=2.328-4.975∆T_{\mathrm{or}}$$

**Figure 14:**
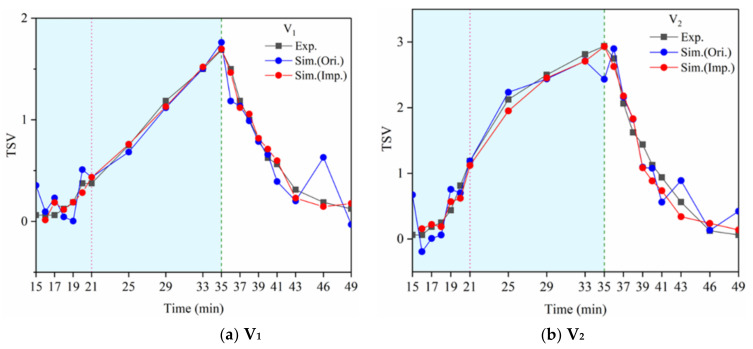
Comparison of the model simulation results.

Figure 14 shows a comparison of the model simulation results from Section 4.1 (original model) and Section 4.4 (improved model) with the experimental data. The original model was found to fit poorly at the beginning of the exercise and the end of the experiment. The improved model fit was better. It indicated that the average change of physiological parameters and their rate of change may have been significant factors in predicting the exercise thermal sensation. Additionally, it was necessary to separate the dynamic and static phases to construct the model better.

### 4.5. Correlation among Subjective Perceptions

Table 7 shows the Pearson correlation coefficient among subjective perceptions. The correlation among subjective perceptions increased with increased exercise intensity. TSV, SFI, and TC were significantly negatively correlated. SFI and TSV were significantly positively correlated, showing that as the thermal sensation increased, the sweaty feeling also increased, while the thermal comfort decreased.

## 5. Discussion

### 5.1. Physiological Indicators around the Dynamic–Static Steps

In the early stages of exercise, the skin temperature decreased slightly and then gradually increases. It can be explained by the thermal balance equation of the human body.

(10) $$M\;-\;W=C+R+E_{\mathrm{sw}}+C_{\mathrm{res}}+E_{\mathrm{res}}+S$$

SHL is defined as sensible heat loss; SHL = C + R. LHL is the loss of latent heat. At the beginning of the exercise, M began to change but did not stabilize immediately. The human body took 5–6 min to reach a new exercising metabolic level [36]. LHL, C_res_, and S did not change significantly in a short time. M-W was reduced, thereby reducing SHL, and SHL reflects the situation of T_sk_. Later, as the metabolic rate increased, the skin temperature gradually rose. This situation can also be explained based on physiology [37,38]. Blood flows to the exercise muscles at the beginning of the exercise to meet the energy requirements of the working muscles, and the blood vessels in the skin begin to contract, causing the skin temperature to drop. In the studies of Zhang et al. [18] and Chappuis et al. [21], it was observed that the core temperature decreased during the initial stage of exercise. This was consistent with our experimental results. Äikäs et al. [39] and Jéquier et al. [40] believe that this fall in temperature was best explained by cooling of central blood due to the mobilization of colder blood coming from the extremities.

Overshoot was observed in skin temperature, oral temperature, SFI, and TSV for a short time after exercise. Because the experiment was performed in a steady-state environment, and the subjects were exercising in place (mainly considering indoor labor or sports), the air velocity brought by the exercise was not significant to LHL. The moment the subject stopped exercising, W stopped immediately, and SHL and LHL did not change significantly in a short time. The metabolic amount did not stop immediately, which can be judged by HR [41]. From the thermal balance equation, it was known that when W = 0, LHL, and SHL remain constant, S was pushed up instantaneously. Due to the increase in heat storage in the body, the core temperature and TSV raised for a short period of time. It made the oral temperature and TSV had overshoots in a short time at the end of the exercise. Simultaneously, to maintain the body’s thermal balance, vasodilation in a short period of time, blood flow increases, resulting in the skin temperature overshoot after exercise. Any factors that may cause vasoconstriction or vasodilatation will affect skin temperature [42]. After exercise, as heat stores increase, the body needs to increase its latent heat loss to maintain its heat balance, thus SFI overshoots.

### 5.2. The Relationship between HRV Indicators and Exercise Thermal Sensation (Comfort)

In the stepwise regression analysis, no significant linear relationship between HRV indicators and TSV was shown. To further investigate the cause, a visual analysis was performed between HRV and TSV. The HRV indicator and its corresponding thermal sensation were sorted out to form a Force-Directed Graph. A Force-Directed Graph, or Force-Based Graph, is used to visualize the connections between objects in a network. By grouping the objects connected to each other in a natural way, a Force-Directed Graph also makes it possible to discover subtle relationships between groups. Each set of experiments was divided into during- and post-exercise, and the TSV rating and the corresponding HRV parameters were statistically classified. The data were divided into three sections. Namely, high-valued section (HS), mid-valued section (MS), and low-valued section (LS), and these values were proportionally calculated. Besides, three value sections were indicated using different colors to visualize the correspondence. Table 8 shows the Force-Directed Graph with the proportional distribution of value sections between HRV and TSV, and the following analysis was obtained:

1. Overall, RMSSD and SDNN distribution trends were similar, with a higher proportion of HS in the during exercise (DE) phase than LS. It indicates that the cardiac load increases during exercise, and the autonomic nervous system were transformed from a state of mutual equilibrium between the sympathetic and vagus nerves at rest to a direction where the sympathetic nerves were dominant [43]. It indicates that the human body was in a state of “excitement”. In particular, in the V_2_ experiment, the proportion of HS was as high as 84.1%, suggesting that increasing exercise intensity has a more significant impact on HRV [44].
2. For time-domain indicators, MS accounts for the highest proportion except for the IE phase of the V_2_ experiment. Under normal conditions, the SDNN and RMSSD values for Chinese were about 3–5 (expressed in natural logarithm transformed values) [45]. The experimental results showed that the time domain indicators returned to standard values after the exercise.
3. SDNN indicators reflect the overall HRV situation. Table 8 shows that both HS and LS decreased, and MS increased after the exercise, indicating a gradual return of HRV to the standard range.
4. As can be seen from the force-directed graph, LS was more likely to be found in the low thermal sensation area. For HS and MS, no significant distribution patterns were observed at the corresponding individual thermal sensation nodes. It also explains, to some extent, the absence of time-domain indicators as an independent variable in the stepwise regression analysis.
5. For the frequency domain indicator (LF/HF), there was almost no LS distribution in the region of the highest thermal sensation node during exercise. It indicates that sympathetic activity was dominant, and the body was in a state of excitement, tension, or discomfort. After exercise, the proportion of LS in the high thermal sensation area increased rapidly, indicating that HF increased, and parasympathetic nerves were activated after exercise, which was consistent with the results of previous studies [44,46]. Parasympathetic nerves were activated, indicating that the “ excitement “ state began to be inhibited, and comfort began to increase. However, as a whole, we still do not observe a more pronounced regular change in the distribution of each value segment in LF/HF with increasing thermal sensation. It may be related to the fact that the body was in exercise and the heart functions were in a complex state.

In summary, changes in cardiac function during and after exercise were complex. It makes changes in the HRV indicator more complicated than in the resting state. In a sense, changes in HRV during and after exercise may be more dependent on changes in body status and consequent changes in heart function. However, the effect on the characterization of thermal sensation and thermal comfort was negligible. Therefore, the desire to analyze exercise thermal sensation using HRV indicators may be difficult to achieve. This analysis was carried out for the reason that the HRV indicator did not enter the multivariate regression equation. Still, it was not possible to prove the existence of other mathematical relationships, which can be further investigated in future studies by attempting to use a computer theory.

### 5.3. Thermal Alliesthesia

In a landmark paper on the physiological role of pleasure, Cabanac [35] coined the term ‘alliesthesia’ for circumstances in which a given stimulus can induce either a pleasant or an unpleasant experience, depending on the subject’s internal state. Richard [47] summarized the simple concept of alliesthesia. He believed that alliesthesia leads us to seek pleasant stimuli and avoid unpleasant ones. Thomas et al. [48] argue that thermal alliesthesia states that the thermal environment’s hedonic qualities were determined as much by the general thermal state of the subject as by the environment itself. In this experiment, it was observed that post-exercise thermal comfort was higher than pre-exercise under the same ambient. We believe that the intensity of exercise governs sensation and delight after exercise rather than not just the ambient conditions. After experiencing thermal discomfort during exercise, subjects experienced an increase in comfort brought about by a decrease of thermal and sweat sensations in a static state, indicating the perception of conditions that could trigger thermal delight, also provide the conditions for thermal alliesthesia. Due to alliesthesia, an asymmetry in thermal comfort before and after exercise was created. Therefore, it is necessary to consider thermal delight and alliesthesia in future thermal comfort studies in exercise. Moreover, we believe that the effect of thermal synesthesia on post-exercise thermal comfort was more delayed or prolonged as the intensity of exercise increases.

### 5.4. Limitations

1. The equations derived from the stepwise regression algorithm can predict thermal sensation and comfort. However, the results obtained using the equations were continuous values, and the answer for each subject is a discrete quantity. Therefore, in follow-up research, we will try to establish a valid threshold range for the results obtained. If the continuous value obtained by the regression equation belongs in this threshold range, the continuous quantity can be converted into an amount discrete that conforms to the ASHRAE thermal sensory scale, making a more accurate prediction of thermal sensation while exercising.
2. However, thermal comfort is a complex topic. The results of this experiment were done at a limited intensity of exercise, and the number of subjects was not large. The common exercise intensities were chosen only as a case study for this work. This work’s results were obtained only under this experiment conditions. Different exercise intensities and times, different temperatures and humidity, and different air velocities can significantly affect physiological parameters and thermal sensations, making significant differences in the results. These results may be valid only for this test case. The authors will add more experimental procedures in future studies to obtain more experimental data.

## 6. Conclusions

This work aimed to analyze the changes and correlations between exercise thermal sensation and physiological parameters in a steady-state environment and evaluate exercise thermal sensation more accurately. We obtained some conclusions consistent with those of previous studies as well as specific conclusions with exercise thermal sensation as follows,

T_sk_, T_or_, TSV, and SFI have transient overshoots after exercise. After a mutation at the metabolic levels (static-dynamic–static), physiological parameters’ adjustment times are symmetrical. Using the average change and rate of change of physiological parameters to predict exercise thermal sensation is a better option. The models should be constructed respectively, according to the dynamic–static state. Additionally, the desire to analyze the exercise thermal sensation using HRV indexes was challenging to achieve. Thermal alliesthesia is a non-negligible factor in the study of exercise thermal comfort.

## Figures and Tables

**Table 1:** Basic information on the subjects.

Gender	No.	Age (Years)	Height (cm)	Weight (kg)	BMI (kg/cm^2^)
Male	9	20.5 ± 2.5	175.1 ± 11.9	66.3 ± 15.7	21.6 ± 3.8
Female	7	20 ± 1	165.6 ± 6.4	59.7 ± 11.3	21.8 ± 5.3
All	16	20 ± 3	170.9 ± 12.1	63.4 ± 18.6	21.7 ± 5.4

Note: BMI is body mass index.

**Table 2:** Measurement instruments of this experiment.

Instrument	Parameter	Measuring Range	Accuracy	Wearing Style
Wireless skin temperature sensor	T_sk_(°C)	10–60 °C	±0.1 °C	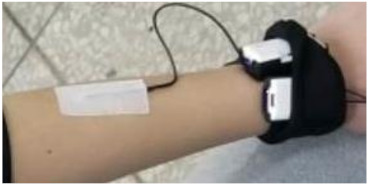
Wireless skin electrodermal activity sensor	EDA(Μs)	0–30 μS	±0.3 μS	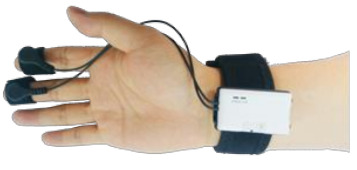
PPG Ear Tip Pulse Sensor	ECG(HR, HRV)	25~240 bpm	±1 bpm	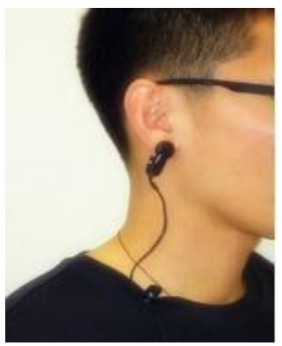

**Table 3:** Stepwise analysis of objective indicators and thermal sensation (TSV).

V_1_	Step 1		Step 2		Step 3		V_2_	Step 1		Step 2		Step 3	
	Coef.	*p*	Coef.	*p*	Coef.	*p*		Coef.	*p*	Coef.	*p*	Coef.	*p*
EDA	0.64	0.004	1.013	0.000	0.846	0.000	EDA	0.409	0.000	0.582	0.000	0.325	0.000
T_or_			−1.277	0.000	−1.299	0.000	T_or_			−1.459	0.001	−2.446	0.000
T_sk_					1.711	0.026	T_sk_					2.189	0.000
R-sq	38.1%		86.2%		90.0%		R-sq	52.2%		77.0%		92.5%	
△R-sq			48.1%		3.8%		△R-sq			24.8%		15.5%	

**Table 4:** Stepwise analysis of objective indicators and thermal comfort (TC).

V_1_	Step 1		Step 2		V_2_	Step 1		Step 2		Step 3	
	Coef.	*p*	Coef.	*p*		Coef.	*p*	Coef.	*p*	Coef.	*p*
T_or_	0.862	0.008	0.967	0.000	EDA	−0.376	0.020	−0.489	0.002	−0.229	0.044
T_sk_			−3.801	0.007	T_or_			1.377	0.016	2.438	0.000
					T_sk_					−2.700	0.005
R-sq	51.8%		79.7%		R-sq	43.2%		71.1%		89.9%	
△R-sq			27.9%		△R-sq			27.9%		18.8%	

**Table 5:** Stepwise analysis of objective indicators and TSV.

V_1_ DE	Step 1		Step 2		V_1_ PE	Step 1		Step 2	
	Coef.	*p*	Coef.	*p*		Coef.	*p*	Coef.	*p*
EDA	1.124	0.000	0.995	0.000	∆Tor	−1.946	0.000	−1.748	0.000
dT_sk_/dt			3.536	0.001	dT_or_/dt			1.025	0.002
R-sq	94.4%		99%		R-sq	91%		98.4%	

**Table 6:** Stepwise analysis of objective indicators and TSV.

V_2_ DE	Step 1		Step 2		V_2_ PE	Step 1	
	Coef.	*p*	Coef.	*p*		Coef.	*p*
∆T_sk_	3.171	0.000	4.255	0.000	∆T_or_	−0.475	0.000
∆T_or_			1.696	0.000			
R-sq	88.2%		98.9%		R-sq	92.1%	

**Table 7:** Pearson correlation coefficient among subjective perceptions.

	TC		SFI	
	V_1_	V_2_	V_1_	V_2_
TSV	−0.530 **	−0.719 **	0.208 **	0.682 **
TC			−0.302 **	−0.528 **

Note: ** denotes *p* < 0.01.

**Table 8:** The force-directed graphs between heart rate variability (HRV) and TSV.

	V_1_						V_2_					
	During Exercise			Post-Exercise			During Exercise			Post-Exercise		
Range of values	＞5 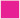 (HS)	3～5 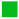 (MS)	2～3 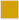 (LS)	＞5 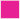 (HS)	3～5 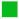 (MS)	2～3 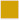 (LS)	＞5 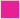 (HS)	3～5 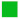 (MS)	2～3 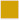 (LS)	＞5 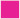 (HS)	3～5 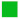 (MS)	2～3 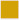 (LS)
Propo -rtion	38.3%	57.9%	3.8%	21.5%	72.0%	6.5%	84.1%	14.3%	1.6%	35.9%	53.0%	11.1%
RMSSD	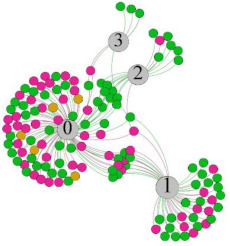			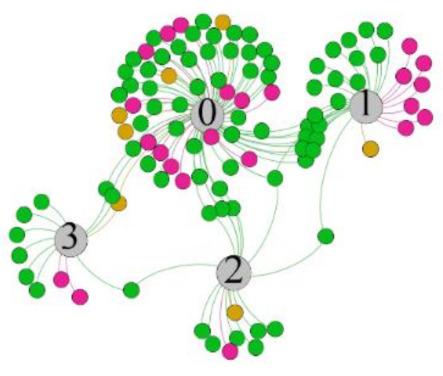			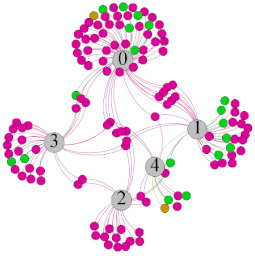			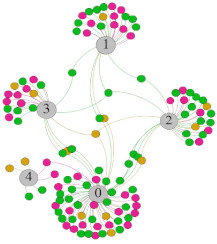		
Propo -rtion	28.3%	58%	13.7%	17.9%	73.2%	8.9%	69.7%	21.2%	9.1%	31.9%	61.2%	6.9%
SDNN	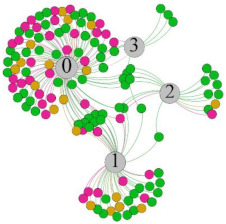			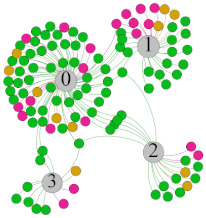			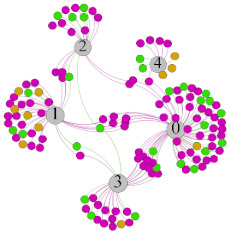			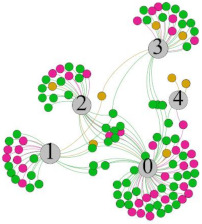		
Range of values	＞3 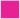 (HS)	1～3 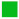 (MS)	0～1 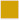 (LS)	＞3 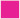 (HS)	1～3 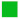 (MS)	0～1 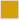 (LS)	＞3 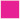 (HS)	1～3 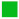 (MS)	0～1 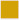 (LS)	＞3 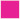 (HS)	1～3 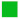 (MS)	0～1 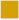 (LS)
Propo -rtion	12.8%	35.2%	52%	13.92%	33.04%	53.04%	13.34%	36.19%	50.47%	23.64%	43.64%	32.72%
LF/HF	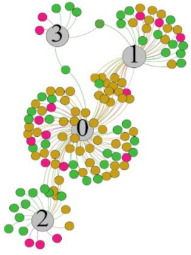			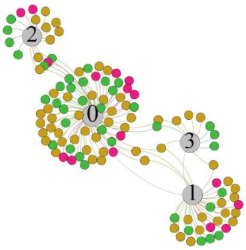			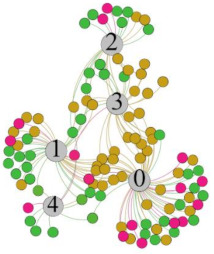			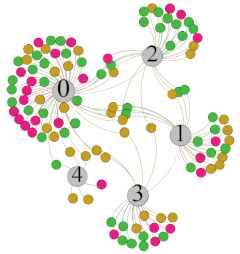		

## Data Availability

The data presented in this study are available on request from the corresponding author. The data are not publicly available due to privacy.

## Nomenclature

T_sk_	mean skin temperature, °C
T_or_	oral temperature, °C
HR	heart rate beats per minute (BPM)
BMI	body mass index
R-R	beat intervals, ms
SDNN	Standard deviation of the NN (R-R) intervals recommended parameters for time-domain measures, the standard deviation of normal sinus R-R interval(take the natural logarithm)
RMSSD	Root mean square of the successive differences; recommended parameters for time-domain tests; the square root of the mean of the sum of the squares of differences between adjacent; beat intervals (take the natural logarithm)
LF	power (0.04–0.15 Hz) in normalized units; LF/(TP-VLF) × 100, n.u.
HF	power (0.15–0.4 Hz) in normalized units; HF/(TP-VLF) × 100, n.u.
LF/HF	ratio LF/HF,
EDA	electrodermal activity, μS
SFI	the sweat feeling index
M	rate of metabolic heat production, W/m^2^
W	rate of mechanical work accomplished, W/m^2^
C	rate of convective heat, W/m^2^
R	rate of radiant heat, W/m^2^
E_sk_	total evaporative heat loss from skin (including natural diffusion E_dif_ and regulative sweating E_sw_), W/m^2^
C_res_	rate of convective heat loss from respiration, W/m^2^
E_res_	rate of evaporative heat loss from respiration, W/m^2^
S	rate of heat storage in the body, W/m^2^
SHL	sensible heat loss
LHL	latent heat loss
